# Hyperosmotic sisomicin infusion: a mouse model for hearing loss

**DOI:** 10.1038/s41598-024-66635-4

**Published:** 2024-07-10

**Authors:** Ayse Maraslioglu-Sperber, Fabian Blanc, Stefan Heller, Nesrine Benkafadar

**Affiliations:** 1 Department of Otolaryngology – Head & Neck Surgery, Stanford University School of Medicine, Stanford, CA 94305 USA; 2 Institute for Stem Cell Biology and Regenerative Medicine, Stanford University School of Medicine, Stanford, CA 94305 USA; 3 Present Address: Department of Otolaryngology – Head & Neck Surgery, University Hospital Gui de Chauliac, University of Montpellier, Montpellier, France

**Keywords:** Cochlea, Hair cell, Supporting cell, Hearing loss, Sisomicin, Apoptosis, Sensory processing

## Abstract

Losing either type of cochlear sensory hair cells leads to hearing impairment. Inner hair cells act as primary mechanoelectrical transducers, while outer hair cells enhance sound-induced vibrations within the organ of Corti. Established inner ear damage models, such as systemic administration of ototoxic aminoglycosides, yield inconsistent and variable hair cell death in mice. Overcoming this limitation, we developed a method involving surgical delivery of a hyperosmotic sisomicin solution into the posterior semicircular canal of adult mice. This procedure induced rapid and synchronous apoptotic demise of outer hair cells within 14 h, leading to irreversible hearing loss. The combination of sisomicin and hyperosmotic stress caused consistent and synergistic ototoxic damage. Inner hair cells remained until three days post-treatment, after which deterioration in structure and number was observed, culminating in a complete hair cell loss by day seven. This robust animal model provides a valuable tool for otoregenerative research, facilitating single-cell and omics-based studies toward exploring preclinical therapeutic strategies.

## Introduction

A major cause of hearing impairment is the loss of cochlear sensory hair cells. Within the cochlear duct are two types of hair cells: inner hair cells are the main transducers of sound stimulation, and outer hair cells are effective amplifiers of mechanical stimulation. Loss of outer hair cells reduces hearing thresholds and frequency tuning, whereas losing inner hair cells severely impedes hearing ability. Profound loss of inner and outer hair cells leads to complete and irreversible hearing loss.

The development of animal models that can reliably replicate cochlear hair cell loss is crucial for advancing effective treatments. Existing strategies typically involve noise exposure or systemic administration of aminoglycosides, often leading to incomplete and non-uniform hair cell loss. This loss is generally most pronounced at the cochlear base and tapers towards the apex^1–11^.

To address these limitations, genetically engineered models such as the *Pou4f3*^*DTR/*+^ mouse have been developed. These mice facilitate nearly complete ablation of cochlear hair cells through cell-specific cytotoxicity^7,12,13^. *Pou4f3*^*DTR/*+^ mice express the human diphtheria toxin receptor gene (*HBEGF*) in a hair cell-specific manner, enabling the elimination of both inner and outer hair cells with a single injection of diphtheria toxin^13–15^. Although highly effective, this model might not fully replicate the complex physiological responses induced by ototoxic drugs, which motivated our study.

Conventional aminoglycoside-induced hair cell loss in vivo requires repeated systemic injections over several days, resulting in asynchronous and incomplete outer hair cell loss^11,16,17^. While a single high dose of aminoglycoside, in conjunction with loop diuretics, administered over several days, can lead to the complete elimination of outer hair cells^2,5,10^, dosing must be carefully controlled to prevent mortality^2^. Furthermore, all these protocols induce asynchronous hair cell loss, with various time points of onset of hair cell demise ranging from forty-eight hours^10^ to four weeks^5^, with low-frequency locations displaying more resilience^6,8^.

Guided by the insights from previous research, we developed a method that combines the efficacy of genetic models with the clinical relevance of aminoglycoside ototoxicity. We employed the aminoglycoside sisomicin to induce controlled hair cell loss in adult mice, mirroring a recent method utilized in an avian model. In chickens, a single infusion of sisomicin into the lateral semicircular canal consistently eliminated all auditory hair cells^18–20^. This technique provided a reproducible pattern of hair cell demise and subsequent hair cell regeneration, and because of the temporal synchrony of hair cell loss, it enabled detailed single-cell RNA-sequencing at specific time points post-infusion. Such sequencing uncovered gene expression changes due to aminoglycoside exposure and subsequent hair cell loss^18,21,22^.

To robustly evoke acute cochlear hair cell loss in adult mice, we infused a hyperosmotic sisomicin solution (ho-sisomicin) into the posterior semicircular canal of four to five-week-old FVB mice. Outer hair cells underwent apoptotic death within a narrow time window of three to eleven hours post-treatment. We also noted a protracted loss of inner hair cells starting at three days post-treatment. Supporting cell numbers remained generally unaffected. Hair cell loss was accompanied by a permanent absence of auditory thresholds observed from three hours post-infusion, based on measuring auditory brainstem responses (ABRs) and distortion product otoacoustic emissions (DPOAEs).

We argue that standardizing experimental conditions using sisomicin across avian and mammalian models will facilitate direct cross-species comparisons and minimize variability. Our protocol provides a uniform and robust method to eliminate cochlear hair cells in adult mice. We did not detect severe cytomorphological changes or loss of other non-sensory cells of the cochlear floor. Because the cochlear floor post-hair cell loss is the target of biological therapies to instigate hair cell regeneration, we envision our model to provide a platform for future systematic studies of the cochlear floor in adult mice upon hair cell loss.

## Results

### Drug infusion into the inner ear

**Figure 1:**
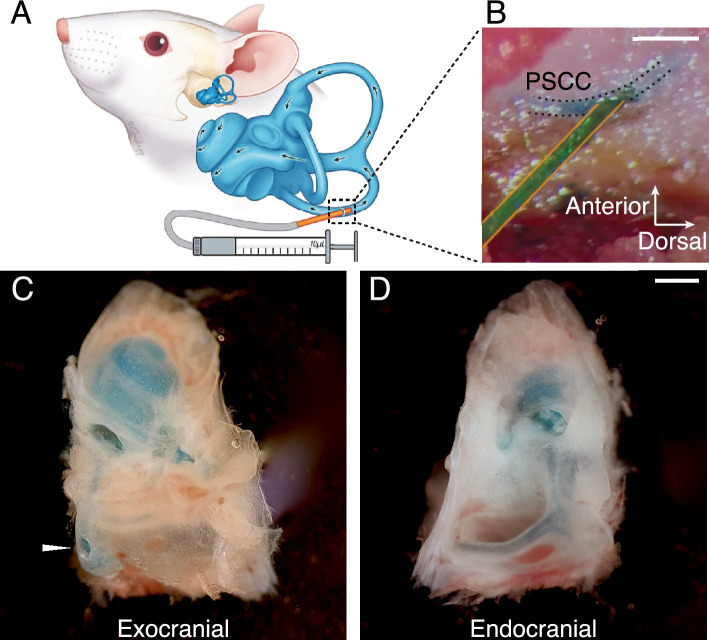
Infusion into the Posterior Semicircular Canal. (**A**) Schematic representation of the posterior semicircular canal (PSCC) infusion installation, with a small polyimide tube (orange) inserted into the bony PSCC lumen connected to a 10 µL syringe and micropump. (**B**) Operative microscope view during the infusion of methylene blue-stained buffer solution into the PSCC. (**C**,**D**) Temporal bone dissection immediately after injection, demonstrating diffusion of the solution throughout the inner ear. The injection site is indicated by the white arrowhead in C. Blue staining is observed in the semicircular canals and all cochlear turns. Scale bars = 500 µm.

We investigated the diffusion of buffer solution into the inner ear of four to five-week-old mice by infusing it into the posterior semicircular canal (Fig. 1A,B). Methylene blue was used to visualize the extent of the solution's diffusion throughout the vestibule and the cochlear perilymph, which predominantly distributes across the scala vestibuli and tympani, with limited presence in the scala media^23^. After injection, the animals were immediately sacrificed, and the temporal bones were dissected (Fig. 1C,D). We found that 1.1–1.5 µL of infused buffer was sufficient for obtaining a distribution of the solution through the semicircular canals and all cochlear turns. Consistent with the observed diffusion path, the efflux of methylene blue exited the cochlear perilymph through the cochlear aqueduct (Suppl. Fig. 1). We concluded that infusion into the posterior semicircular canal facilitates drug diffusion throughout the inner ear, including the full tonotopic range of the cochlea.

### Sisomicin treatment and serendipitous discovery of the effects of hyperosmolality on hair cells and supporting cells

We next infused the aminoglycoside sisomicin into the left semicircular canal of postnatal day (P) 28 mice. Sisomicin, a minor constituent of the multi-componential gentamicin complex, is produced with high purity by its manufacturer, ensuring consistent and reliable experimental outcomes. It is notably ototoxic, selectively targeting sensory hair cells, which facilitates the controlled induction of hair cell loss^24–27^. This property, along with its high level of ototoxicity, makes sisomicin particularly useful for time-sensitive experiments and the study of acute hair cell damage effects^18^.

**Figure 2:**
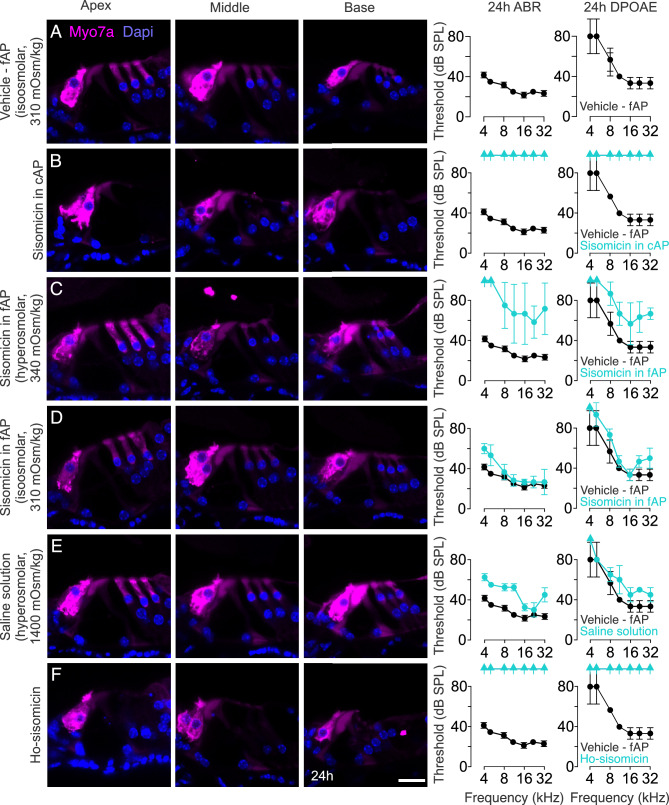
Synergy of Hyper-Osmolality and Sisomicin. (**A**) Shown are confocal images of organ of Corti transverse vibratome sections 24 h after infusion of freshly prepared artificial perilymph (fAP) (vehicle fAP control). Normal histology (no hair cell loss) is apparent along the cochlear duct from the apex, middle, and base. On the right, ABR and DPOAE thresholds are shown (black). This control baseline measurements for ABR and DPOAE are used for comparison with all other experimental conditions (**B**–**F**). (**B**) Infusion of 20 mg/mL sisomicin in commercially procured artificial perilymph (cAP) resulted in the complete loss of outer hair cells from apex to base, accompanied by a loss of ABR and DPOAE thresholds (cyan; N = 3), compared with the vehicle fAP control measurements (black), which are also shown in (**A**). (**C**) Infusion of 20 mg/mL sisomicin in fAP, not adjusted for osmolality and therefore slightly hyperosmolar. A few outer hair cells in the base were lost after 24 h, accompanied by a shift of ABR and DPOAE thresholds (cyan; N = 3). (**D**) 20 mg/mL sisomicin in fAP with adjusted osmolality elicited minimal ABR threshold shifts and no hair cell loss (cyan; N = 3). (**E**) Injection of hyperosmotic saline solution resulted in a few missing outer hair cells in the basal turn and a slight ABR and DPOAE threshold shift (cyan; N = 3). (**F**) The combination of 20 mg/mL sisomicin and hyperosmotic saline solution resulted in complete outer hair cell loss in the base and middle parts of the cochlea, with some remaining outer hair cells in the apex, and no detectable ABR and DPOAE thresholds (cyan; N = 3). ABR and DPOAE thresholds of the vehicle fAP control cochlea are shown in black in panels (**B**–**F**) for reference. The arrows indicate a complete lack of response at the highest stimulus level. Scale bar = 20 µm.

To ensure the infusion procedure itself did not cause damage, we used a vehicle injection of freshly prepared artificial perilymph (fAP) or physiological saline solution and observed no hair cell loss, with mice displaying normal ABR and DPOAE thresholds 24 h after injection (Fig. 2A, Suppl. Fig. 2A). This confirmed the safety of the infusion procedure, consistent with previous reports^28–30^ and uninjected control ears (Suppl. Fig. 2B).

To maintain consistency and minimize variability, we decided to use a commercial preparation of artificial perilymph (cAP). Infusing sisomicin in cAP resulted in complete outer hair cell loss and no detectable ABR or DPOAE thresholds at 24 h post-treatment (Fig. 2B). Concurrently, we tested sisomicin in freshly made artificial perilymph (fAP), observing no hair cell loss but a noticeable shift in ABR and DPOAE thresholds at the same 24-h time point (Fig. 2C).

Puzzled by this inconsistency, we contacted the manufacturer for details about the cAP preparation but obtained unsatisfactory responses. To investigate further, we measured the osmolality of the 1X cAP and discovered it to be extremely hyperosmotic at approximately 5000 mOsm/kg. Becoming aware of the possible importance of infusion buffer osmolality^31^, we proceeded to measure the osmolality of the 20 mg/mL sisomicin in fAP, which revealed a mild hyperosmolarity (340 mOsm/kg). Subsequently, we carefully prepared an iso-osmolar solution of 20 mg/mL sisomicin in fAP (310 mOsm/kg). This adjusted preparation caused only a small ABR threshold shift at low frequencies and did not induce any hair cell loss (Fig. 2D). In contrast, the hyperosmotic cAP solution led to significant hair cell loss and auditory dysfunction, as shown in Fig. 2E.

Our next objective was to determine whether osmolality shift alone or the combined effect of both factors was essential for inducing outer hair cell depletion. To investigate this, we chose 0.9% normal saline, which by itself did not cause hair cell loss or affect hearing fidelity^32^ (Suppl. Fig. 2A). We hypothesized that saline presents the advantage of having the simplest composition of solutions for inner ear infusion. We manipulated osmolality by adding mannitol to create a 1,400 mOsm/kg hyperosmolar saline solution, which resulted in small ABR and DPOAE threshold shifts and the loss of a few outer hair cells in the basal turn of the cochlea (Fig. 2E). Infusion of 20 mg/mL sisomicin in a 500 mOsm/kg hyperosmolar saline solution produced a similar result (Suppl. Fig. 2C). Finally, the infusion of 20 mg/mL sisomicin diluted in saline solution with added mannitol to achieve a final osmolality of 1,400 mOsm/kg (ho-sisomicin) replicated the results of the infusion of 20 mg/mL sisomicin in cAP. It led to an almost complete outer hair cell loss (Fig. 2F) and an absence of DPOAE thresholds (Fig. 2F), indicating that a combination of high osmolality and sisomicin induces profound outer hair cell death. The profound ABR threshold shifts suggest that inner hair cells are also compromised, contributing to the observed severe hearing loss.

**Figure 3:**
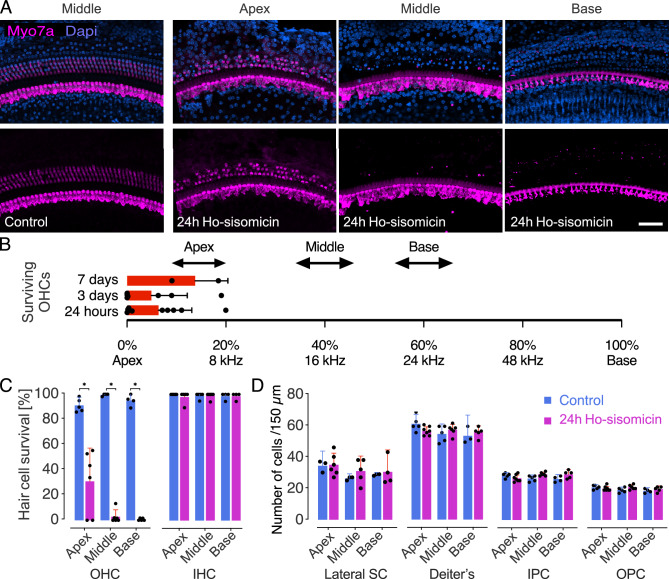
Ho-sisomicin Treatment Ablates Outer Hair Cells after 24 h. (**A**) Wholemounts labeled with antibodies to Myo7a to reveal inner and outer hair cells. The right ear control cochlea was unaffected by ho-sisomicin infusion into the left ear’s posterior semicircular canal. Three rows of outer hair cells and a single row of inner hair cells were visible in all turns; the middle section is shown. At 24 h post-ho-sisomicin treatment (PhoST), all outer hair cells in the base and middle were ablated; some animals showed surviving outer hair cells in the apex. Inner hair cells were not affected. (**B**) Surviving outer hair cells (OHCs) are located in the most apical region of the cochlea. This was consistent after 24 h, 3 days, and 7 days PhoST. Each dot represents a different cochlea PhoST and is located at the transition between the region of partial surviving OHCs and the more basal complete OHCs loss. The arrowed lines represent the location of the sections shown in (**A**). (**C**) Quantification of surviving hair cells 24 h post-infusion revealed a significant loss of OHCs. Inner hair cells (IHC) were not affected. (**D**) We counted supporting cell nuclei in the different subtypes of cochlear supporting cells, which we identified by their distinct location and cytomorphology. This analysis revealed no significant difference between the PhoST and control groups. Bar plots show the mean with standard deviation as whiskers. Each dot represents a single count from a 200 µm portion of the apex, middle, or base of one cochlea. *SC* supporting cells, *IPC* inner pillar cells, *OPC* outer pillar cells. In each subgroup, N = 3–8 cochleae (combining control and experimental groups). Each dot in the plot represents data from one animal. Statistical significance for the survival of OHCs at the apex, middle, and base of the cochlea was assessed using a two-way ANOVA followed by Holm-Sidak's multiple comparisons test. The significant *p*-values obtained are 0.0043 for the apex, 0.0095 for the middle, and 0.0159 for the base, respectively. Scale bar = 100 µm.

Whole-mount immunohistochemistry of the cochlea confirmed an almost complete loss of outer hair cells at 24 h post-treatment with ho-sisomicin (Fig. 3A). None of the assessed animals had more than 20% surviving outer hair cells in the extreme apical region, and 44% exhibited complete outer hair cell loss (Fig. 3B and Suppl. Fig. 3A). Quantitative analysis revealed a significant reduction in the number of surviving outer hair cells, primarily concentrated in the middle and basal regions of the cochlea (Fig. 3C). We did not detect significant differences in the numbers of inner hair cells and the various subtypes of cochlear supporting cells between the ho-sisomicin solution and control groups at 24 h post-treatment (Fig. 3C,D and Suppl. Fig. 3A,B). The number of supporting cells also remained unchanged at 48 h and seven days post-treatment (Suppl. Fig. 4).

These results suggest a synergistic effect between sisomicin and hyperosmolality in specifically targeting the outer hair cells without an immediate effect on inner hair cell numbers and on supporting cells.

### Time course of ho-sisomicin-induced outer hair cell death

**Figure 4:**
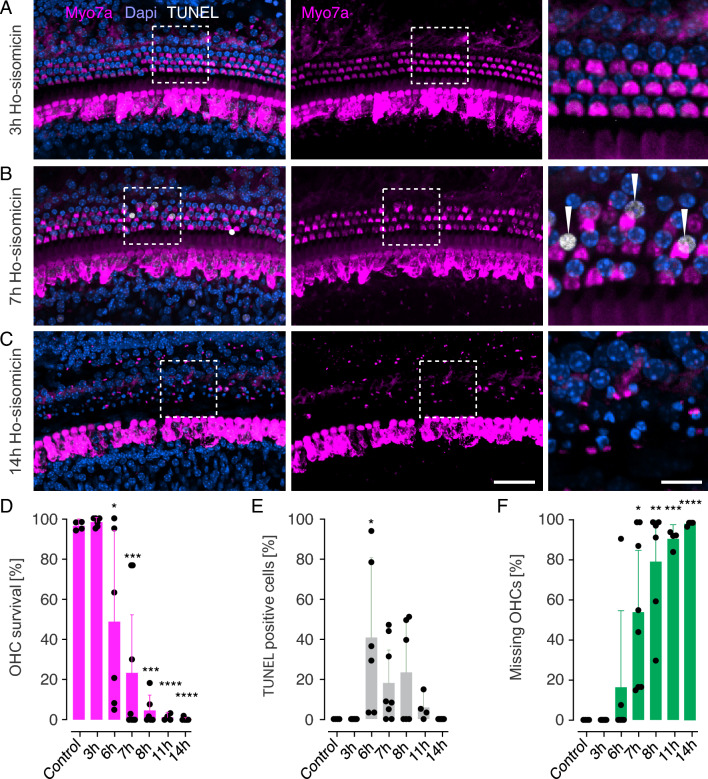
Rapid Outer Hair Cell Death via Apoptosis. (**A**) At 3 h post ho-sisomicin treatment (PhoST), the cochlear sensory epithelium shows normal morphology without TUNEL-positive nuclei. (**B**) TUNEL-positive outer hair cell nuclei were visible at 7 h PhoST, while no TUNEL-positive supporting cell or inner hair cell nuclei were detected. (**C**) At 14 h PhoST, complete outer hair cell loss with surviving inner hair cells and supporting cells was observed. Some Myosin 7a staining remained in the outer hair cell region, associated with hair cell debris. The examples shown in A-C are from a middle region of the cochlear duct. (**D**) Quantification of surviving outer hair cells (OHC) shows a significant decrease starting at 6 h PhoST, with complete OHC loss observed at 11–14 h PhoST. The significant *p*-values are: 0.0142 (6 h PhoST); 0.0006 (7 h PhoST); 0.0001 (8 h PhoST); < 0.0001 (11 h PhoST); < 0.0001 (14 h PhoST). (**E**) Quantification of TUNEL-positive OHC nuclei shows that dying hair cells are in late-stage apoptosis from 6 h to 11 h PhoST. The majority of TUNEL-positive OHCs was observed at 6 h PhoST. The significant *p*-value is 0.0358 for the 6-h time point. (**F**) Gradual increase in missing OHCs or OHC corpses from 6 h PhoST onward. The significant *p*-values are: 0.0463 (7 h PhoST); 0.0016 (8 h PhoST); 0.0001 (11 h PhoST); < 0.0001 (14 h PhoST). Bar plots show the mean with standard deviation as whiskers. Each dot represents a count from a 200 µm portion of the middle or base of one cochlea. Left scale bar = 50 µm, right scale bar: 10 µm; N = 3–6 animals for each time point. Holm-Sidak’s multiple comparisons test was applied to determine statistical differences between the control group and each subsequent time point.

We next performed experiments to investigate the time course and the mechanism of ho-sisomicin-induced outer hair cell death at different time points post-treatment using terminal deoxynucleotidyl transferase dUTP nick end labeling (TUNEL). At 3 h post-ho-sisomicin treatment, no TUNEL-positive nuclei were observed, and the sensory epithelium appeared normal (Fig. 4A). However, at 7 h post-ho-sisomicin treatment, we detected TUNEL-positive outer hair cell nuclei while no TUNEL-positive inner hair cell and supporting cell nuclei were visible (Fig. 4B). TUNEL detects nuclear DNA fragmentation, which is a hallmark of apoptosis, and it indicates that the labeled cells are in the final stages of programmed cell death. By 14 h post-ho-sisomicin treatment, we observed a complete loss of outer hair cells with surviving inner hair cells and supporting cells (Fig. 4C). Some Myosin 7a immunoreactivity, likely associated with outer hair cell debris, remained in the outer hair cell region (Fig. 4C).

Quantification confirmed the histological observations and revealed a significant decrease of outer hair cells at 6 h post-ho-sisomicin treatment and complete outer hair cell loss after 14 h (Fig. 4D). TUNEL-positive outer hair cell nuclei peaked at 6 h post-treatment, indicating that most cells enter the late stages of apoptotic cell death between 3 and 6 h after exposure to ho-sisomicin. DNA fragmentation in outer hair cells was evident between 6- and 11- hours post treatment (Fig. 4E). Moreover, the number of missing outer hair cells and outer hair cell debris increased gradually, starting from 6 h post-ho-sisomicin treatment (Fig. 4F).

Our results indicate that ho-sisomicin treatment induces a rapid and selective death of outer hair cells through apoptosis. In contrast, inner hair cells and supporting cells appear unaffected within the first 24 h.

### Delayed inner hair cell death

Previous studies have demonstrated that aminoglycosides preferentially target outer hair cells, and inner hair cells generally remain unaffected^5,8,33^. Consistent with these findings, we observed no significant changes in the number of inner hair cells 24 h after ho-sisomicin injection, despite the near-complete loss of outer hair cells (Fig. 3A,C).

**Figure 5:**
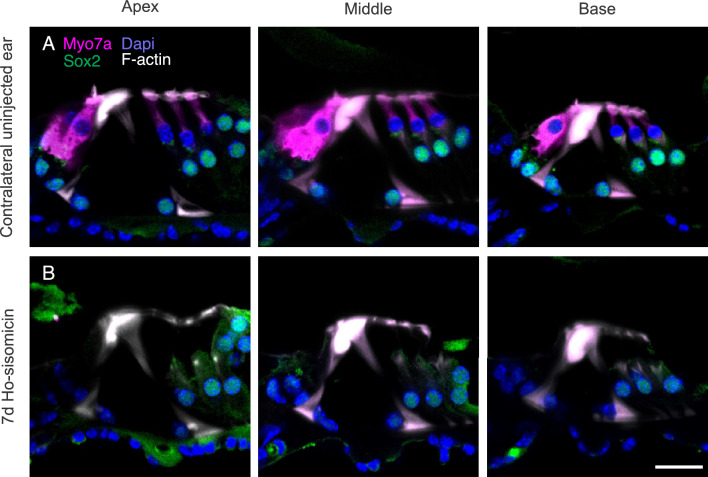
Profound Inner Hair Cell Loss 7 Days Post-ho-sisomicin Infusion. (**A**) Right ear control cochlea at 7 days PhoST, showing the presence of three rows of outer hair cells and a single row of inner hair cells in magenta (Myosin 7a). (**B**) In the ho-sisomicin infused left cochlea, all inner hair cells were lost at 7 days PhoST. The F-actin-rich pillar cells remained unaffected. Scale bar = 20 µm.

To explore possible long-term effects, we examined the fate of inner hair cells beyond the initial 24-h post-treatment period and waited for seven days after ho-sisomicin infusion. While the contralateral right uninjected ear showed no damage at seven days (Fig. 5A), over 70% of ho-sisomicin-infused cochleae displayed a consistent and complete loss of inner hair cells (Fig. 5B). We did not encounter animals without inner hair cell loss but rather with a more extensive cell loss. For example, on some occasions, the delayed damage of the organ of Corti after seven days included supporting cells (Suppl. Fig. 5A). Moreover, one animal exhibited a flat epithelium seven days after the ho-sisomicin infusion (Suppl. Fig. 5B). These observations emphasize the inherent in vivo variability that needs to be acknowledged as a limitation of the method employed in our study. An evaluation of the individual surgical infusions suggests that the main cause of variability could be the placement of the infusion needle and different levels of leakage during drug administration. With the delivered volume set to a specific parameter, such as 1.1 µL, the actual volume delivered into the posterior semicircular canal can vary between surgeries. This variability in the infused volume likely contributes to the observed variation in results. We estimate that each experimenter will ultimately have a sweet spot that defines the average absolute infused volume of the drug solution in each experiment.

**Figure 6:**
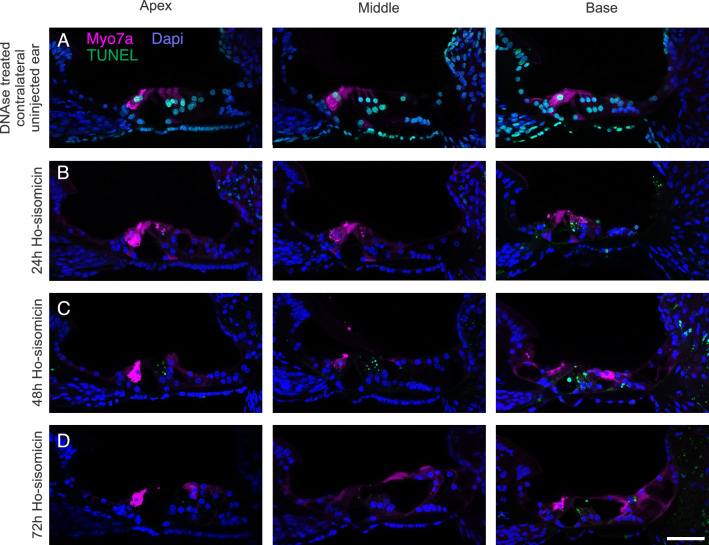
Ho-sisomicin-Induced Inner Hair Cell Death is not Caused by Apoptosis. (**A**) DNAse treated contralateral uninjected ear shows normal cochlear epithelium morphology with three rows of outer hair cells and a single row of inner hair cells in magenta. A brief treatment with DNAse was used to create fragmented DNA as a positive control for TUNEL (green nuclei). (**B**,**C**) 24 h and 48 h post ho-sisomicin treatment, inner hair cells were not affected. Some TUNEL-positive outer hair cell debris is visible. (**D**) At 72 h post-injection, inner hair cell death was visible. The remaining inner hair cells have TUNEL-negative nuclei. Scale bar = 50 µm; N = 3 cochleae at each time point.

We wanted to understand further when and how inner hair cells die. To do so, we subjected the samples to TUNEL at 24, 48, and 72 h post ho-sisomicin infusion. We treated the contralateral uninjected ear with DNAse as a technical control for TUNEL (Fig. 6A). At 24 and 48 h post ho-sisomicin infusion, all examined cochleae displayed intact inner hair cells with normal cytomorphology (Fig. 6B,C, and Suppl. Fig. 6A,C–E). However, at 72 h post-infusion, we observed some loss of inner hair cells (Fig. 6D and Suppl. Fig. 6B). Interestingly, we did not detect TUNEL-positive inner hair cell nuclei at any investigated time point (Fig. 6 and Suppl. Fig. 6A,B). These results suggest that the delayed loss of inner hair cells within the observed time frame is not associated with apoptosis.

Our findings show that combining sisomicin with hyperosmolarity results in a relatively consistent impact on inner hair cells, resulting in their death, albeit delayed with respect to outer hair cell loss. Furthermore, our study demonstrates that this phenomenon is not limited to inner hair cells alone but also involves the potential loss of supporting cells in certain instances. The observed inconsistent and asynchronous cellular demise underscores the intricate nature of this process within an in vivo context.

### Structural changes in the *stria vascularis*

**Figure 7:**
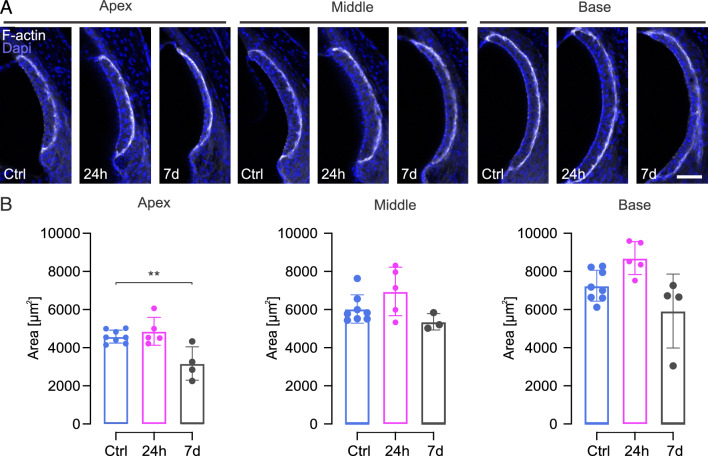
Structural Changes in the *Stria Vascularis* after Ho-Sisomicin Infusion. (**A**) Vibratome sections of controls (N = 8) and cochleae 24 h (N = 5) and 7 days (N = 4) post ho-sisomicin infusion. Cell nuclei and F-actin are fluorescently labeled with Dapi and phalloidin. Scale bar = 50 µm. (**B**) Quantification of the stria’s cross-sectional area revealed a mild swelling of the stria in the base and middle regions of the cochlea after 24 h. Seven days post ho-sisomicin treatment, we detected a significant decrease of the stria’s cross-sectional area in the apex with a *p-*value of 0.0067). Bar plots show the mean with the standard deviation shown as whiskers. Statistical significance was assessed using a two-way ANOVA followed by Holm-Sidak’s multiple comparison test.

Ototoxic trauma, particularly noise but also aminoglycosides^4,10,34^ do not only affect the organ of Corti but also other cochlear structures. The *stria vascularis* is a physiologically essential structure in the lateral wall of the cochlea. Its function is ion homeostasis and generation of the endocochlear potential in the *scala media*. We measured the cross-sectional area of the *stria vascularis* at 24 h and seven days post-ho-sisomicin treatment, comparing it with the contralateral uninjected ear (Fig. 7). The cross-sectional area was determined through F-actin labeling of the tissue that revealed the stria’s histomorphology (Fig. 7A).

Quantification indicated a non-significant increase of the cross-sectional area in the middle and basal part of the cochlea 24 h post-infusion. Seven days following the infusion, a notable reduction was observed, with significance confined to the apical region (Fig. 7B).

Our findings suggest that the damage induced by ho-sisomicin extends beyond the cochlear epithelium and also impacts the *stria vascularis*. Notably, similar changes in the *stria vascularis* are observed in other damage paradigms, such as those induced by noise, indicating that changes in the *stria vascularis* are a common response to various types of cochlear insult^35^. However, in our study, it remains to be determined whether these effects are due to sisomicin alone or in combination with the high osmolality of the solution.

### Ho-sisomicin treatment causes permanent hearing loss

**Figure 8:**
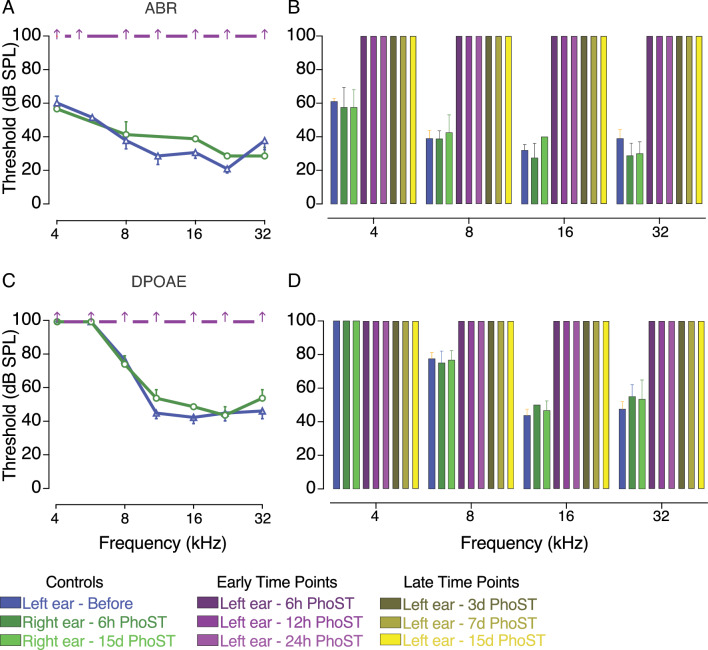
Ho-sisomicin Treatment Induces Rapid Hearing Loss. (**A**) Auditory Brainstem Response (ABR) thresholds in pre-infusion control (blue) and contralateral post-infusion control (dark green) inner ears show normal hearing thresholds. Six hours post-ho-sisomicin treatment (PhoST, dark magenta), no ABR thresholds were detectable. (**B**) Controls and additional time points reveal the permanent loss of ABR thresholds. (**C**) Distortion Products of Otoacoustic Emissions (DPOAE) thresholds obtained before ho-sisomicin treatment (blue) or from contralateral controls (dark green) show normal DPOAE thresholds. No thresholds were detected six hours PhoST. (**D**) Controls and additional time points reveal the permanent loss of DPOAE thresholds. Bar plots show the mean with the standard deviation represented as whiskers, with each plot including data from 3 to 4 animals per time point. Holm-Sidak’s multiple comparisons test was applied to evaluate statistical differences across all time points for threshold losses in ABR and DPOAE. Results show that all threshold losses were highly significant (*p* < 0.0001), while all control groups showed no significant differences (*p* > 0.1).

The administration of ho-sisomicin significantly impacted the cochleae of adult mice, leading to rapid and irreversible hearing loss. Six hours post-treatment, we observed a complete absence of ABR and DPOAE thresholds across all frequencies in injected left ears (Fig. 8). The ABR waveforms recorded from the affected left ears showed prolonged latency and responses only to stimuli above 70 dB SPL, accompanied by signal crosstalk from the contralateral ear^36^. However, following the surgical destruction of the right ear, these ABR waveforms disappeared, and no signals were recorded for stimuli less than 100 dB SPL. We classified the 100 dB SPL thresholds as "no threshold" values for the tested frequencies in the left ear. Thus, our findings support the conclusion that ho-sisomicin infusion into the posterior semicircular canal enables effective drug diffusion throughout the cochlea, resulting in rapid apoptotic outer hair cell loss accompanied by immediate and permanent hearing loss, ultimately ablating all hair cells.

## Discussion

Hearing loss affects hundreds of millions worldwide, necessitating the development of novel therapeutic interventions to prevent or even restore cellular loss and cochlear function in patients. The loss of cochlear hair cells is the major primary contributor to permanent hearing loss. To effectively develop treatments, it is crucial to use animal models in which hair cell loss can be induced reliably. Hair cells are the most sensitive component of the mammalian cochlea and can be eliminated with acoustic overstimulation or ototoxic drugs such as aminoglycoside antibiotics. Despite this susceptibility, ablating hair cells in the mouse cochlea using loud noise or systemic administration of aminoglycosides is a challenging endeavor^7^. The shortcomings of many existing drug-based damage models are likely owed to the fact that the effective drug concentration inside the cochlear duct is insufficient to kill the more drug-resistant apical outer hair cells and inner hair cells.

Our study aimed to address the challenges of creating a reliable mouse model for hair cell loss by using a chemically defined ototoxic compound, sisomicin, infused into the posterior semicircular canal^18^. We found that the infusion of sisomicin in a hyperosmotic solution leads to rapid and nearly complete outer hair cell loss, with a corresponding absence of ABR and DPOAE thresholds. This hair cell loss is selective for outer hair cells within the first 24 h, with delayed inner hair cell loss occurring between days 3 and 7. The use of a hyperosmotic solution is crucial for achieving effective hair cell ablation, as iso-osmolar solutions did not produce similar results.

The high osmolality likely enhances the ototoxic effects of sisomicin by increasing its uptake or potentiating its toxic effects on hair cells. This synergistic relationship mirrors the mechanism observed in systemic aminoglycoside ototoxicity models, where loop diuretics enhance drug efficacy^10^. Osmolality shifts may affect ion pumps and channels, fluid volumes, and the endocochlear potential, potentially contributing to hair cell damage. High osmolality could disrupt ionic homeostasis, increasing cell stress and thresholds for apoptosis. Further investigation is needed to explore these mechanisms and their contribution to the observed hair cell loss.

The immediate and extensive outer hair cell loss observed in our study contrasts with the gradual decline seen in aging or milder ototoxic events. This rapid loss suggests an initial selectivity of ho-sisomicin for outer hair cells. This selective impact aligns with previous studies demonstrating the preferential targeting of outer hair cells by aminoglycosides. The loss of hair cells was sudden and extensive, with evidence suggesting a minimal role for macrophages or supporting cells in immediate debris clearance.

The delayed inner hair cell loss observed between days 3 and 7 post-treatment without corresponding apoptotic indicators may result from secondary effects. The rapid loss of outer hair cells is unprecedented and could disrupt the cochlear microenvironment, leading to progressive inner hair cell damage. The involvement of different mechanisms in the demise of inner and outer hair cells warrants further investigation. Understanding why inner hair cells exhibit a longer timeline of degeneration and a lack of obvious apoptotic markers compared to outer hair cells could provide insights into the differential susceptibility and response of these cell types to ototoxic insults.

It was surprising that we detected a complete absence of ABR and DPOAE thresholds across all frequencies already three hours after the ho-sisomicin infusion. If only outer hair cells were initially impacted by the infusion, we would observe an absence of DPOAE thresholds but a moderate ABR threshold shift. However, the immediate absence of thresholds on both evaluations was detected before the death of outer and inner hair cells. This observation suggests that the initial compromise of cochlear function is more complex than simply causing the death of hair cells. Cytomorphologically, inner hair cells appeared intact at least for the first 48 h after treatment. Still, we cannot exclude latent physiological effects on the electrochemical signaling components of intact-appearing hair cells and other structures in the cochlea. Our data suggest that inner hair cells are impacted, and examination seven days after ho-sisomicin infusion revealed the complete loss of inner hair cells. We detected some variability in a small proportion of specimens that manifested in more severe damage, such as supporting cell loss or even epithelial flattening. While variability exists, the method is generally reliable and results in the complete ablation of all cochlear hair cells after 7 days.

One important aspect to consider is the broader implications of our findings for understanding drug toxicity and osmolality effects in the cochlea. The synergistic effect between sisomicin and hyperosmolality in specifically targeting initially outer hair cells followed by inner hair cells highlights the potential for developing more effective ototoxicity models. This could lead to better screening methods for otoprotective drugs and more accurate assessments of ototoxic risk in clinical settings. Moreover, our model provides a valuable platform for studying the molecular and cellular mechanisms underlying hair cell death and regeneration, offering insights that could guide the development of regenerative therapies.

Additionally, our findings raise questions about the potential effects of hyperosmolality on other cochlear structures and functions. For instance, osmolality shifts might impact ion transport mechanisms, cause temporal fluid volume changes, and could affect the endocochlear potential, which are critical for normal hearing function. Investigating these effects could provide a more comprehensive understanding of how changes in the cochlear environment contribute to hair cell damage and hearing loss.

The observed temporal decoupling of cellular demise, with outer hair cells dying rapidly via apoptosis while inner hair cells exhibit delayed death, underscores the complexity of hair cell pathology. This finding highlights the need for further research to elucidate the mechanisms driving outer and inner hair cell loss. It also emphasizes the importance of considering the temporal dynamics of hair cell death when developing therapeutic strategies.

Despite some variability in the extent of damage, our method offers a reliable model for inducing synchronized hair cell loss across the cochlea. This synchronicity allows for detailed investigation of gene expression changes in dying hair cells and surviving cochlear cells, providing a valuable platform for developing regenerative therapies. Comparing this model to the *Pou4f3*^*DTR/*+^ mouse model, which uses diphtheria toxin for hair cell ablation, could yield insights into different mechanisms of hair cell loss and potential therapeutic targets.

In conclusion, our findings demonstrate that infusing sisomicin in a hyperosmotic solution into the posterior semicircular canal is an effective method for unilaterally inducing rapid and complete outer hair cell ablation, followed by inner hair cell demise, with broader implications for understanding drug toxicity, osmolality effects, and hair cell regeneration strategies. Despite these open questions, we argue that the method has an applied advantage: its synchronicity and the uniformity of its impact across the entire cochlea. As we have previously shown for the avian inner ear^18,21,22^, we expect that the synchronicity, and especially the two distinct waves of hair cell loss, will be useful to determine gene expression changes in dying hair cells and surviving cochlear floor cells but also provide a robust platform for developing and testing new therapeutic interventions aimed at preventing or reversing hearing loss.

## Methods and materials

### Animal surgery

Male and female FVB mice aged 4 to 5 weeks (Charles River Laboratories, St-Constant, QC) were used. The Stanford University Institutional Animal Care and Use Committee approved the housing and animal procedures. All methods were performed in accordance with the relevant guidelines and regulations, including adherence to the principles outlined in the ARRIVE guidelines.

Mice were anesthetized with 2 L/min oxygen and 2% isoflurane using a nose cone. During the surgery, the body temperature of the mice was maintained at 37.5 °C utilizing a heating pad.

The postauricular area was shaved, and the skin was disinfected using alternating 10% povidone-iodine (Betadine, Purdue Products, Stamford, CT, Cat #301013-OC) followed by 70% ethanol for three times. The mice received subcutaneous administration of 300 µL of carprofen (5 mg/kg, Rimadyl, Zoetis, Parsippany, NJ) for analgesia. The animals were confirmed to exhibit no reflex to painful stimulation before surgery.

Under sterile conditions, a left post-auricular incision was made. The sterno-cleïdo-mastoïd muscle was bluntly dissected, exposing the posterior part of the temporal bone and the square angle formed by the lateral and posterior semicircular canals. A canalostomy of the posterior semicircular canal was performed using a 26G beveled needle. Effusion of lymph confirmed the opening of the endosteum of the bony canal. The hole was then widened using a micro-driller (Performance Micro Tool, Cat #100M2X300S) to approximately the size of the outer diameter of the polyimide tube (124 µm, Microlumen, 039).

The procedure involved inserting a polyimide tube into the posterior semicircular canal, which was sealed to the temporal bone using two drops of tissue adhesive (3 M Vetbond, St. Paul, MN) to prevent leakage. The polyimide tube was attached to a PVC tube (Tygon) connected to a 10 µL Hamilton syringe. A total of 1.1 µL of sisomicin or control solution (for details, see “Drug preparation”) was infused into the posterior semicircular canal at a rate of 4 nL/s using a Micropump (UMP3 Micro 4, World Precision Instruments).

After the injection, the tube was left in place and was closed by melting the free tip with an electrocautery pen (Thermo Fisher Scientific, Cat #NC9721806). Special care was taken to avoid any leakage at the injection site during and after the injection. The skin was closed in two layers with surgical glue. The total duration of the procedure was approximately 30 min. The isoflurane was reduced to 0% for recovery, and pure oxygen was provided at 4 L/min on a heating pad until the animal moved normally. After the injection, the animals were monitored daily. They presented moderate signs of left vestibular deficit (head tilt, circling behavior) during the first two days after injection. These symptoms were compatible with normal activity and feeding and improved in the following days.

### Drug preparation

Methylene blue at a concentration of 10 mM, prepared in 1X PBS, was initially used as the buffer solution. This dye was utilized to visualize the diffusion of the solution throughout the inner ear, following its infusion into the posterior semicircular canal of four to five-week-old mice. The sisomicin solution was prepared by dissolving sisomicin (MedChemExpress, Cat #HY-B1222) at a concentration of 20 g/L in sterile 0.9% sodium chloride solution or artificial perilymph. To increase the solution's osmolality, mannitol (Sigma-Aldrich, Cat #M4125-100G) was added and thoroughly mixed until completely dissolved. The amount of mannitol required to achieve the desired osmolality varied depending on the initial osmolality of the sisomicin solution and the desired final osmolality. Osmolality was assessed using a vapor pressure osmometer (Wescor Vapro 5520) to ensure accurate measurement. Two types of artificial perilymph were utilized in this study. The first type was purchased from Biochemazon (Cat #BZ285), while the second type was prepared in-house in accordance with a previously described method^37^.

A dose–response curve was used to determine the appropriate osmolality of the sisomicin solution to achieve the desired hair cell damage. High osmolality sisomicin (ho-sisomicin) was freshly prepared immediately before each injection for a group of 3 animals.

To ensure the accuracy and validity of our results, we included control groups in our study. The first control group underwent a sham treatment, which involved an infusion of sterile 0.9% sodium chloride solution or artificial perilymph without adding sisomicin. The second control group received a dose of iso-osmolar sisomicin solution. These control groups were essential for comparison and establishing the specific effects of the hyperosmotic sisomicin solution used in our experimental group.

### Functional hearing assessment

To assess the effect of the ho-sisomicin infusion on the function of hair cells, we conducted ABR and DPOAE recordings before and at different time points after surgery (6 h, 14 h, 24 h, three days, seven days, and 15 days). All functional assessments were performed under anesthesia using ketamine (100 mg/kg) and xylazine (10 mg/kg) in a Faraday-shielded, anechoic, sound-proof chamber. We maintained the body temperature at 37.5 °C with the help of a heating pad.

#### Distortion product of otoacoustic emissions

DPOAEs were recorded in the external auditory canal using a calibrated probe. The two primary tones of frequency f1 and f2 were generated with a constant f2/f1 ratio of 1.2, geometrically centered around the tested frequency, and the distortion product 2f1-f2 was processed by the RZ6 Processor and visualized by BioSigRZ software (Tucker-Davis Technologies). The f1 and f2 were presented simultaneously, and the ratio remained the same, from 100 dB SPL to 20 dB SPL in 10 dB decrements. We tested 4, 5.7, 8, 11.3, 16, 22.6, and 32 kHz. For each tested frequency, the threshold was defined as the lowest intensity where a distortion product 2f1-f2 emerged more than 20% over the neighboring noise.

#### Auditory brainstem responses

Auditory Brainstem responses were recorded using the RZ6 Processor and BioSigRZ software (Tucker-Davis Technologies). Electrodes were placed subcutaneously on the vertex, beneath the ear, and in the leg of anesthetized mice, connected to a RA4PA preamplifier and RA4LI head stage. Tone bursts of 10 ms duration were delivered at a rate of 21/s at 4, 5.7, 8, 11.3, 16, 22.6, and 32 kHz. Sounds were provided by a Tucker-Davis loudspeaker in a calibrated closed-field condition. Amplification of responses from the electrodes was achieved with a Nexus type 2690 amplifier (Bruel and Kjaer) and averaged 512 times. Level-amplitude functions of the ABRs were obtained at each frequency by varying the level of the tone bursts from 0 to 80 dB SPL in 5 dB decremental steps. ABR threshold was defined as the lowest sound level at which a reproducible waveform could be observed. For the right ear, after the initial complete set of frequencies recorded before injection, only four frequencies (4, 8, 16, 32 kHz) were tested to assess the contralateral effect of the left ear injection.

### Dissection of sensory organs and vibratome sectioning

The heads were bisected and the bony ear capsules were removed using surgical scissors (Cat #F.S.T. 14002-13). The tissue was then fixed in 4% paraformaldehyde in PBS overnight at 4 °C, following which the cochleae were either briefly stored in 1 X PBS at 4 °C or directly processed. For vibratome sections, cochleae were decalcified in 0.5 M EDTA (Thermo Fisher Scientific, Cat # AM9261) for 3 to 4 days. To maintain tissue structure during sectioning, the cochleae were embedded in 4% low melt agarose in disposable molds (Cat #VWR 15160-215). Embedded specimens were stored at 4 °C in a humidified chamber. The cochleae were sectioned transversely using a Leica VT1200 vibratome (100 µm thickness, 1 mm amplitude, 0.6 mm/sec speed) in cold 1X PBS. The resulting sections were decanted onto a Sylgard^®^ 184 silicone plate, and excess agarose was removed by punching out round cochlear sections with a 1.5 mm biopsy punch.

### Immunohistochemistry

Immunocytochemistry was performed in cochlear whole-mount preparations and on anatomical vibratome sections. We used anti-Myosin 7a (1/500, Proteus Biosciences, Cat #25-6790) and Sox2 (1/50, Santa Cruz Biotechnology, Cat #sc-365823). Rhodamine-conjugated phalloidin (1/2000, Thermo Fisher Scientific, Cat #A22287) was used to label F-actin. Secondary antibodies were diluted at 1:500. These included donkey anti-mouse and anti-rabbit, respectively conjugated to Alexa 488 and Alexa 568 (Thermo Fisher Scientific, Cat #A-21202 and Cat #A10040). DNA was stained with Dapi (1/1000 MilliporeSigma, Cat #D3571). DeadEnd™ fluorometric TUNEL System was used to identify apoptotic DNA fragmentation (Promega, Cat #G3250). DNAse was used as a positive control to demonstrate the efficacy of the TUNEL staining in the cochlear epithelium. After labeling, we mounted the sections on glass slides in antifade medium (Citifluor CFM-3; Electron Microscopy Sciences, Cat #17979-30) with a 0.12 mm spacer (Thermo Fisher, Cat #S24735) between the slide and the coverslip. We conducted primary antibody omission controls to ensure that secondary antibodies did not produce specific labeling. All experiments were performed at least in triplicate, and representative images are shown.

### Imaging and quantitative analysis

Confocal microscopy was performed using a Zeiss LSM700 microscope at 1.0 zoom and 20X or 40X magnification (Plan-Apochromat, Numerical Aperture 1.3, oil immersion), and images were acquired using Zen Black software. To analyze the confocal stacks and generate maximum intensity projections, ImageJ software (NIH) was used. Transverse sections are presented as confocal projections of < 10 slices of 0.5–1 µM Z-depth. To quantify the number of cells per independent experiment, at least three 100 × 100 µm quadrants were randomly selected on whole mount preparations at the cochlea’s basal, middle, and apical regions. Cell counts for cells labeled with Myosin 7A and Sox2 antibodies were performed in these images using the Cell Counter plug-in for Fiji software. At least three independent experiments were performed for each data point to ensure the accuracy and reproducibility of the results.

### Quantification and statistical analysis

Cell counts were exported and graphically displayed in GraphPad Prism software. Significant differences between groups were assessed with two-way ANOVA followed by Holm-Sidak’s multiple comparisons test. Significant *p*-values are indicated in the legends of each figure.

### Ethics declarations

All the animal experiments were performed by the ARRIVE guidelines and approved by the Stanford Institutional Animal Care and Use Committee (IACUC) under protocol # 33189.

### Supplementary Information

Supplementary Figures.

## Data Availability

All data generated or analyzed during this study are included in this published article and its Supplementary Information. The source data are provided with the paper.
