# Supplementary material for: Hyperosmotic sisomicin infusion: a mouse model for hearing loss
Source: Sci Rep. 2024 Jul 10;14:15903. doi: 10.1038/s41598-024-66635-4 (PMC11237112; doi:10.1038/s41598-024-66635-4)
Supplement: Supplementary file 1 — Supplementary Figures. [file 41598_2024_66635_MOESM1_ESM.docx]

**Supplementary Figures**

**Suppl. Figure S1, related to Figure 1:**

**
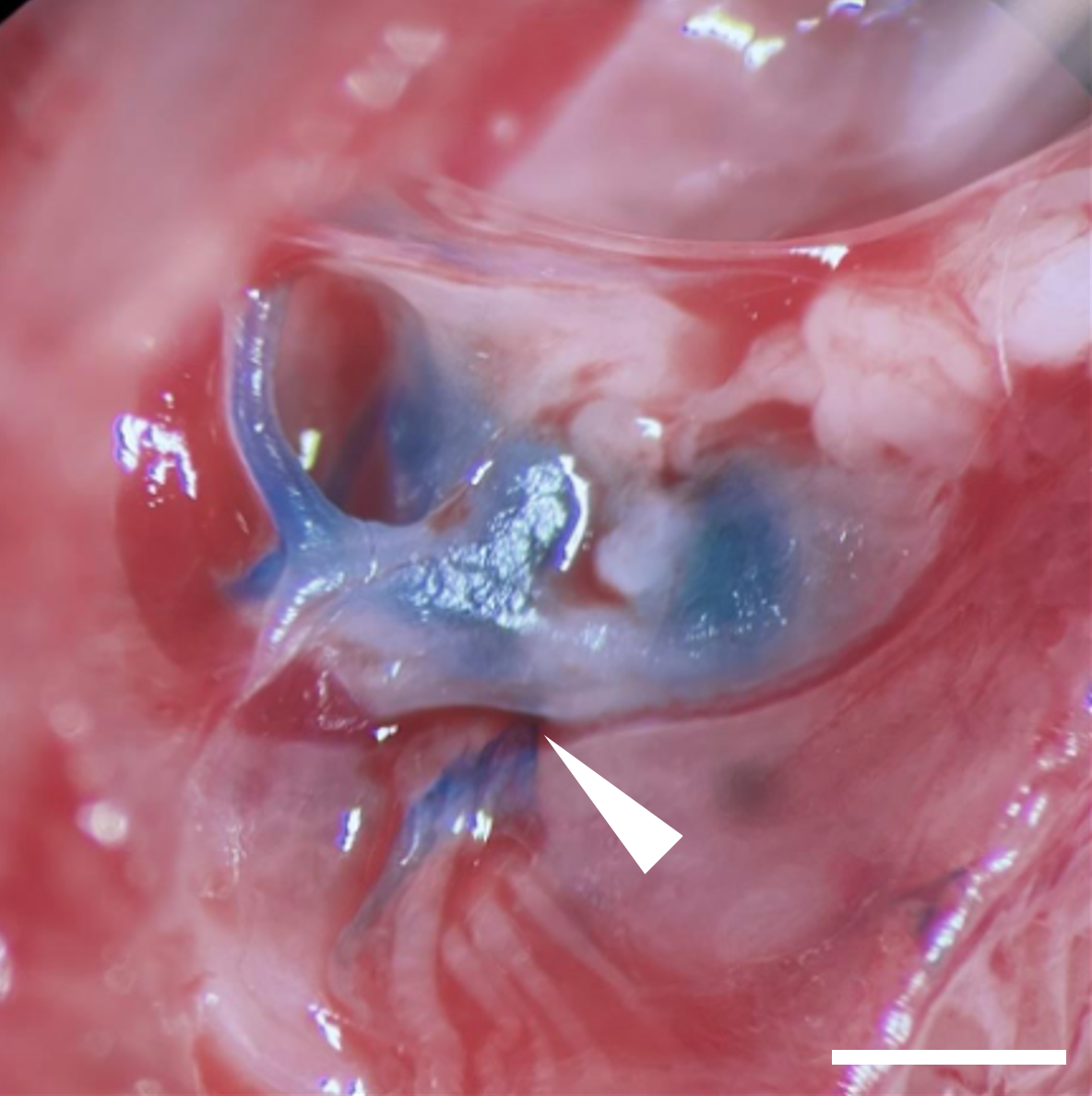
**

Intracranial observation of the inner ear immediately after infusion of 1.5 µL of methylene blue through the posterior semicircular canal. Complete coloration of the perilymph fluid of the inner ear can be seen, with the efflux of methylene blue exiting the inner ear through the intracranial opening of the cochlea aqueduct (white arrow). Scale bar = 1 mm.

**Suppl. Figure S2, related to Figure 2:**

**A.** 24 hours after infusion of saline solution, normal histology (no hair cell loss) is apparent in transverse vibratome sections from the apex, middle, and base. Normal ABR and DPOAE thresholds (cyan; N = 2) were measured and not different from vehicle fAP control measurements.

**B.** Confocal images from a control left ear without any kind of injection showing normal morphology with one row of inner hair cells and three rows of outer hair cells. ABRs and DPs were measured before infusion (cyan; N = 5) and are not different from vehicle fAP control measurements.

**C.** Infusion of 20 mg/mL sisomicin in hyperosmolar (500 mOsm/kg) saline showed little outer hair cell loss (an example with a full complement of hair cells is shown), accompanied by a shift of ABR and DPOAE thresholds (cyan; N = 3).

ABR and DPOAE thresholds of the vehicle fAP control (N = 3) cochlea are shown in black in panels B-F for reference. ABR and DPOAE plots show the mean with the standard deviation as whiskers. Scale bar = 20 µm.

**Suppl. Figure S3, related to Figure 3:**

**A.** Two examples of the observed variability of outer hair cell loss in the apex of the cochlea. Wholemount preparations show the apex of two cochleae 24 hours after ho-sisomicin infusion. Left: Cochlea with surviving apical outer hair cells corresponding to the 6 kHz region. Right: Cochlea exhibiting complete loss of outer hair cells. Note the presence of a single row of inner hair cells in both cochleae. Scale bar = 100 µm.

**B.** Presence of supporting cells 24 hours post-infusion. Wholemount preparations comparing a control cochlea with a cochlea 24 hours post-infusion. The three rows of outer hair cells are absent, while the single row of inner hair cells remains after infusion (labeled in magenta, Myosin 7a). The supporting cell nuclei, labeled with Sox2 (in green), are still present, although a disorganization of the supporting cells in the outer hair cell region is observed. Scale bar = 50 µm.

**Suppl. Figure S4, related to Figure 3:**


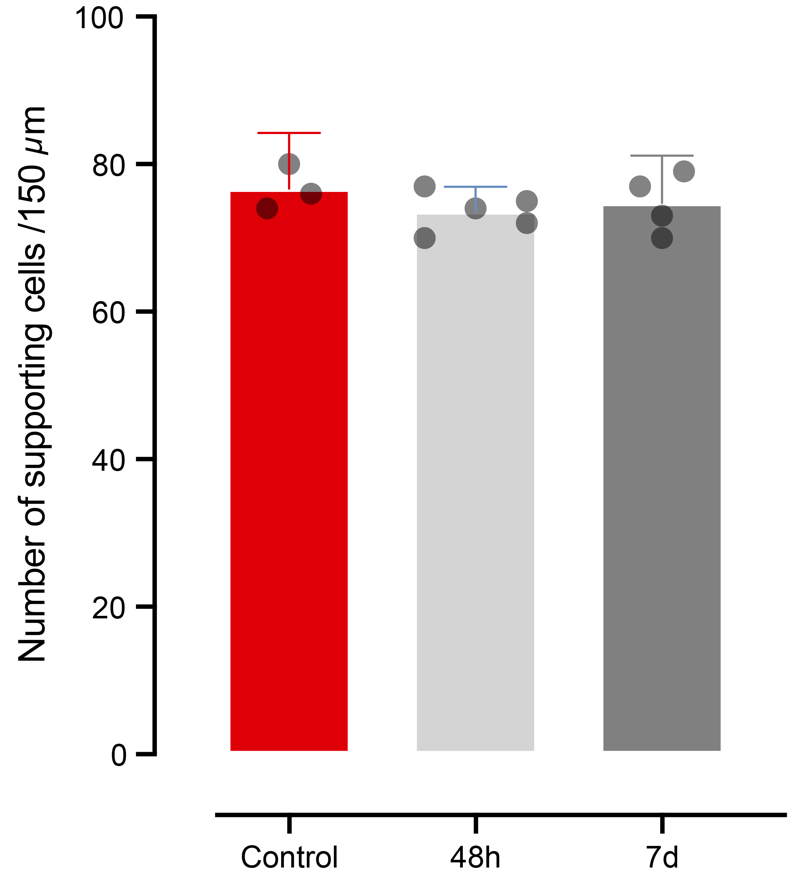


Quantification of supporting cell numbers 48 hours and seven days post-infusion revealed no statistically significant changes compared to the control. Analysis was conducted on N = 3 cochleae per time point. For the 48-hour and 7-day time points, two different cochlear segments were analyzed from two cochleae, resulting in five data points per time point.

**Suppl. Figure S5, related to Figure 5:**

**A, B.** Examples of profound cochlear cell loss observed in a few post-ho-sisomicin cochleae after 7 days; middle turn cross-sections are shown. Supporting cells are lost in (A) and a flat epithelium is visible in (B).

Scale bar = 20 µm.

**Suppl. Figure S6, related to Figure 6:**

**A, B.** Wholemount preparations of cochleae 48h (A) and 72h (B) post ho-sisomicin infusion showed some missing inner hair cells and disturbed inner hair cell cytomorphology at 72 h (B). The remaining inner hair cells do not show TUNEL labeling. Left scale bar = 50 µm, right scale bar: 10 µm.

**C, D, E.** Wholemount preparations of control cochleae (C), and 24h (D) and 48h (E) post ho-sisomicin infusion showing normal IHC hair bundles. IHCs appear unaffected at 24h but show some aberrations at 48h post-treatment.

Left scale bar = 10 µm, right scale bar: 50 µm.
